# Pattern Recognition of Pyrolysis Bio-Oils by GC×GC-TOFMS with Tile-Based Feature Selection and Principal Component Analysis

**DOI:** 10.1021/acsmeasuresciau.5c00061

**Published:** 2025-08-25

**Authors:** Anna Clara de Freitas Couto, Marília Gabriela Pereira, Wenes Silva, Tarcísio M. Santos, Jhonattas C. Carregosa, Julian E. B. Castiblanco, Jandyson Machado Santos, Alberto Wisniewski, Leandro Wang Hantao

**Affiliations:** † Instituto de Química, Universidade Estadual de Campinas, Campinas 13083-862, Brasil; ‡ Instituto Nacional de Ciência e Tecnologia (INCTBio), Campinas 13083-862, Brasil; § Departamento de Química, Universidade Federal Rural de Pernambuco, Recife 52171-900, Brasil; ∥ Grupo de Pesquisa em Petróleo e Energia de Biomassa, Departamento de Química, 74391 Universidade Federal de Sergipe, São Cristóvão 49100-000, Brasil

**Keywords:** chemometrics, bio-oil, agro-industrial biomass, pyrolysis, lignocellulosic biomass

## Abstract

Chemometrics associated with advanced analytical separation methods are crucial for the chemical profiling of complex samples, such as bio-oil, enabling more accurate and efficient identification of differential features. The composition of bio-oils influences the selection of pretreatment methods for fuel production, which may include processes such as filtration, guard bed usage, or reactions such as hydrothermal liquefaction and esterification. This study focuses on the chemical profiling of pyrolytic bio-oils from sugar cane bagasse and straw using comprehensive two-dimensional gas chromatography coupled with time-of-flight mass spectrometry (GC×GC-TOFMS). Chemometric approaches such as tile-based Fisher ratio analysis (FRA) and principal component analysis (PCA) are employed for the feature selection of class-differentiating analytes. Bio-oils from both feedstocks exhibited chromatographic profiles with subtle differences, which were observed in the composition and relative abundance of specific compound classes. Bagasse bio-oil was rich in phenolics and hexose derivatives, such as furans and aldehydes. In contrast, straw bio-oil presented a higher abundance of hydrocarbons and fatty acid methyl esters. Tile-based FRA enabled the identification of 16 differential features and the detection of low-intensity compounds, such as long-chain esters and hydrocarbons, not previously detected by the peak table-based approach. PCA based on these differential features explained 98.7% of the total variance (PC1 + PC2), clearly grouping bio-oils by feedstock origin. The findings highlight the potential of GC×GC-TOFMS and chemometrics for differentiating bio-oils, demonstrating the importance of advanced analytical techniques in studying biomass conversion processes and characterizing bioproducts.

## Graphical abstract

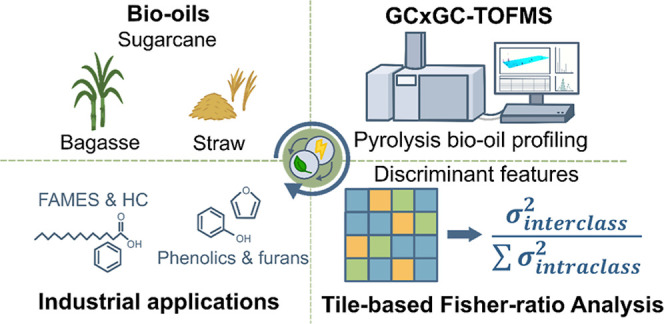

## 1 Introduction

Lignocellulosic bio-oil has emerged as a promising alternative to petrochemicals as efforts to transition to renewable energy sources are encouraged globally.^1,2^ Its potential stems from the wide variety of naturally occurring organic compounds found in the biopolymers of plant-based biomasses such as lignin, cellulose, and hemicellulose. Sugar cane agricultural residues, such as bagasse and straw, represent an abundant and inexpensive biomass source for biofuel and, specifically, bio-oil production.^3^ In 2024, the worldwide production of sugar cane reached 2.9 trillion tons, from which 27% refers to bagasse and 14% to straw, both residues from commodity processing.^4–6^

Physical, chemical, or biochemical conversion procedures are applied to extract chemicals of interest from lignocellulosic biomass efficiently. As a thermochemical conversion, pyrolysis thermally decomposes biopolymers into smaller fragments in an inert atmosphere, producing bio-oil, a highly complex organic matrix.^7^ The bio-oil composition is significantly influenced by pyrolysis conditions, including temperature, heating rate, residence time of the vapors produced, and the distribution of lignocellulosic content within the biomass.^8^ For example, lignin decomposes mainly into phenolics of multiple levels of complexity, while glucose in the cellulose chain decomposes into pyrans and furans, and hemicellulose produces mostly oxygenated linear compounds.^9^

Comprehensive two-dimensional gas chromatography coupled with time-of-flight mass spectrometry (GC×GC-TOFMS) has been recently employed to characterize bio-oil more extensively.^10–13^ Its enhanced peak capacity reduces peak overlap, providing a more accurate chemical profiling of such complex mixtures.^14^ Previous studies successfully applied GC×GC to characterize sugar cane straw bio-oils and found phenolics to be the main product.^15,16^ Additionally, another report observed hydrocarbons and less concentrated hexose derivatives exclusively when analyzing sugar cane straw and bagasse bio-oil using comprehensive two-dimensional gas chromatography coupled with mass spectrometry (GC×GC–MS). Consequently, these findings suggest that GC×GC–MS may be the ideal technique for the qualitative analysis of complex bio-oils, enabling a 3-fold improvement in the number of analytes successfully identified, compared with one-dimensional gas chromatography coupled to mass spectrometry (1D-GC-MS).^17^

GC×GC–MS analysis often results in a complex and information-rich data matrix, comprising a third-order data tensor, i.e., a chromatographic profile with the first (^1^D) and second (^2^D) dimensions as well as the mass spectra of each pixel. Therefore, chemometric methods are necessary to process and extract relevant chemical information from multivariate data efficiently. For exploratory, discovery-based analysis, one can perform untargeted analysis by evaluating all features that may be responsible for the differences observed between the classes of samples. Unsupervised pattern recognition, such as principal components analysis (PCA), may be used to highlight such intrinsic differences between classes. However, important information may be overshadowed by unwanted interclass variations.^18^ To overcome this problem, the use of feature selection methods, such as Fisher ratio analysis (FRA), can aid in selecting the class-distinguishing features from the data set. The FRA uses the Fisher ratio (F-ratio) by dividing the class-to-class variance (σ_class‑to‑class_^2^) and the pooled within class variation of a specific feature (σ_within‑class_^2^) (eq 1). FRA software generates a list of features organized by the descending F-ratio value. A feature with a high F-ratio value is more likely to be statistically different in intensity between the classes evaluated.^19^

1 $$Fisher‐ratio=\frac{\sigma_{class‐to‐class}^{2}}{\sum \sigma_{within‐class}^{2}}$$

Therefore, the greater the F-ratio value, the greater the discriminant power of a variable. Many studies have successfully applied the FRA in analysis ranging from biological to fuel samples, indicating the potential of the method for identifying differential features for class discrimination.^20–24^

Retention time shifts are inherent to chromatography and require feature alignment strategies for adequate data processing. Marney et al.^25^ introduced the tile-based FRA algorithm to mitigate retention time misalignment without explicitly aligning the data. Instead of working with peak table-based or pixel-based approaches, the raw chromatographic data are binned and summed within defined tiles across the two-dimensional space, before calculating the F-ratios. The dimensions of the tile inherently incorporate a tolerance range that accounts for minor retention time shifts in both dimensions. The tile-based approach minimizes the potentially adverse impact of pixel misalignment. Additionally, the demand for computational resources and time is minimized.^19^ As a result, the tile-based FRA generates a table of features ranked by their statistical relevance for class discrimination, which can be subjected to a spectral library search for chemical identification. With careful evaluation and preselection strategies,^26^ the analyst can identify tiles with the most meaningful chemical information.

Chemometric methods and approaches are routinely employed for petroleomics analysis.^23,24,27–29^ Similarly, as a complex matrix with potential as a sustainable biofuel or fuel additive, employing chemometrics strategies could contribute to chemical profiling of pyrolytic bio-oils and fuel development efforts.^30,31^ Therefore, this study aims to apply the FRA with a tile-based approach to explore the chemical composition of sugar cane bagasse and straw pyrolytic bio-oil analyzed by GC×GC-TOFMS and determine differential features for biomass discrimination. This compositional information determines the selection of pretreatment methods for fuel production, which may include processes such as filtration, guard bed usage, or reactions such as hydrothermal liquefaction and esterification.

## 2 Materials and Methods

### 2.1 Sample Preparation

Sugar cane bagasse and straw were collected in Iracemápolis (São Paulo, Brazil), sundried to a humidity content below 10%, and ground to homogeneous particles smaller than 1 mm. Immediate analysis (moisture, organic matter, and ash) and elemental analysis (C, H, N, and O) were performed. 100 mg of each biomass was pyrolyzed in a microscale pyrolytic reactor with a heating rate of approximately 250 °C min^–1^ to 500 °C and a residence time of 30 s. The procedure was conducted under constant nitrogen flow, as described elsewhere.^32^

### 2.2 Bio-Oil Characterization through GC × GC-TOFMS

The bio-oils were dissolved in 5 mL of tetrahydrofuran (THF, CAS no. 109-99-9). Aliquots of 300 μL were derivatized with 40 μL of MTBSTFA (*N*-*tert*-butyldimethylsilyl-*N*-methyltrifluoroacetamide) with 1% *t*-BDCMS (*tert*-butyldimethylchlorosilane, CAS 77377-52-7).^33^ The derivatization step occurred at 70 °C with constant agitation for 5 min. True replicates were prepared (*N* = 3).

A GC×GC-TOFMS system was used to analyze the samples. The system consisted of an Agilent 8890 gas chromatograph coupled to a Pegasus BT 4D time-of-flight mass spectrometer (Leco Corporation, St. Joseph, Michigan, USA). An Agilent 7650 autosampler was used to inject 0.3 μL of the sample. The inlet was kept at 300 °C with a split ratio of 30:1. The column set was nonpolar × midpolar with the ^1^D being a Rxi-5MS column (30 m × 0.25 mm × 0.25 μm) (Restek Corporation—Bellefonte, PA, USA). A Rxi-17SilMS column (2.0 m × 0.15 mm × 0.15 μm; Restek Corp., Bellefonte, PA, USA) was used as the ^2^D column. A two-stage QUADJET thermal modulator was used with a modulation period of 8 s. The oven temperature was set to 100 °C, with a heating rate of 3 °C min^–1^ to 240 °C, followed by 25 °C min^–1^ until 300 °C. The modulator and second oven had a constant +15 °C offset from the first oven. The transfer line and ion source temperatures were set to 300 and 250 °C, respectively. Helium was used as the carrier gas at 1.0 mL min^–1^ flow. Electron ionization was performed with 70 eV. Mass spectra were acquired from 40 to 500 Da at a rate of 50 Hz.

### 2.3 Data Processing

LECO ChromaTOF software (version 1.2, LECO Corporation, St. Joseph, MI, USA) was applied to process data acquired by GC×GC-TOFMS. Parameters for peak table-based processing, regarding peak finding and spectra generation, were a signal-to-noise ratio (S/N) of 15 and a minimum stick count of 15.

ChromaTOF Tile software (version 1.01, LECO Corporation, St. Joseph, MI, USA) was used to analyze significant differences between sample classes through tile-based F-ratio analysis.^26^ The tile size and time shift were determined and optimized from the original data, by measuring the length and observed shifts in retention time for 1D and 2D of a common peak across replicates and samples, as described by Cain et al.^18^ The signal-to-noise ratio (S/N) was set to 10, and a F-ratio threshold of 700 was applied.

Putative identification of mass spectra from peak table-based and tile-based approach was performed with spectral library search (NIST—USA National Institute of Standards and Technology, Gaithersburg, MD, USA, version 2.4, 2024) with a minimum of 80% similarity match for identification level 2 and 3, according to the proposed minimum reporting standards of Metabolomics Standards Initiative.^34^ A tolerance of 50 units was used for retention index filtering for identification level 2. PCA and figures were generated with R statistical computing software (version 4.4.0).^35^

## 3 Results and Discussion

### 3.1 GC×GC-TOFMS Characterization of Sugar Cane Bagasse and Straw Bio-Oils

Physico-chemical analysis of sugar cane bagasse and straw was performed. The results for elemental analysis, moisture content, and organic matter are presented in Table 1. It can be readily seen that bagasse exhibits a higher percentage of organic matter (91.8%) compared to straw (84.1%). This suggests that both sugar cane bagasse and straw can produce significant amounts of bio-oil; however, the highest yield is expected for sugar cane bagasse. Elemental analysis reveals a considerable amount of oxygen in bagasse (50.4%) and straw (45.9%), indicating that bio-oils should exhibit large concentrations of oxygenated compounds. To verify these observations, a detailed composition analysis of sugar cane bagasse and straw bio-oils was performed using GC×GC-TOFMS.

**Table 1:** Immediate and Elemental Analysis of Sugarcane Bagasse and Straw

biomass	bagasse (wt %)	straw (wt %)
Immediate Analysis		
moisture	6.1	8.4
organic matter	91.8	84.1
ash	2.1	7.5
Elemental Analysis		
C	43.4	46.3
H	5.3	7.3
N	0.9	0.5
O	50.4	45.9

Using this instrument configuration, the analytes are separated based on volatility (vapor pressure) as the ^1^D uses a nonpolar stationary phase. The separation in the ^2^D is based on specific interactions with the midpolar stationary phase (polarity). Therefore, a structured chromatogram can be observed in Figure 1, showing a clearly ordered elution pattern of peaks that belong to similar chemical groups.

**Figure 1:**
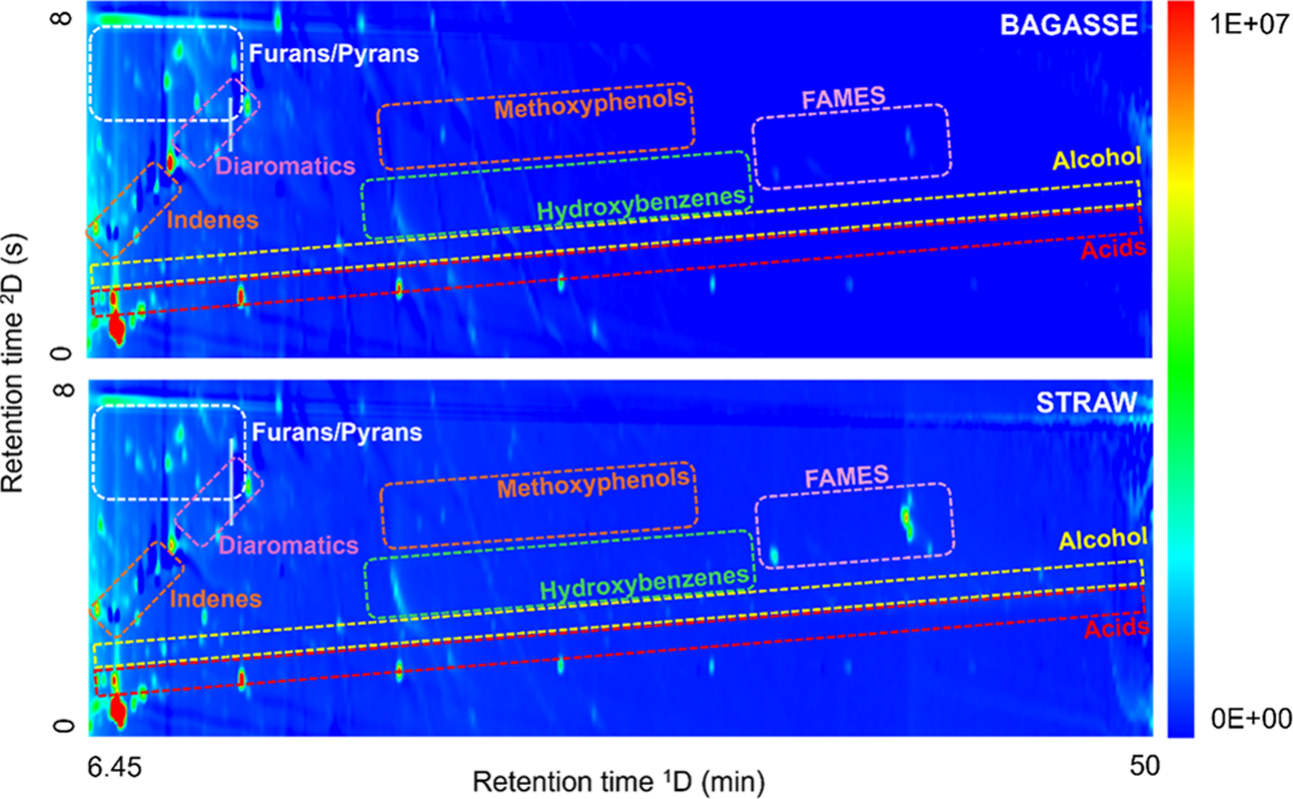
Total ion current chromatograms (TIC) of sugar cane bagasse and straw bio-oils obtained by GC×GC-TOFMS, with highlights to major chemical groups such as furans/pyrans, diaromatics, indenes, methoxyphenols, hydroxybenzenes, fatty acid methyl esters (FAMEs), alcohols, and acids.

Qualitative analysis based on mass spectral library search and retention index filtering enabled the identification of 236 compounds for sugar cane bagasse and 268 compounds for straw bio-oil, corresponding to 27.2% and 29.4% of all detected areas, respectively. The elution pattern was used to validate the identity of the peaks (Table S1). It is estimated that the lower coverage arises from the intrinsic limitations of using mass spectral library searchers as the primary tool for analyte identification as well as constraints in deconvoluting coeluted peaks at lower concentrations. For instance, a previous study focused on the characterization of sugar cane straw bio-oil using GC×GC-TOFMS, which identified at least 32% of the sample.^15^

The chemical class distribution is shown in Figure 1, with the relative areas corresponding to the peak area divided by the sum of the areas of all identified peaks. Although the response factor of each compound is not considered, a semiquantitative analysis of the percentage of peak area and qualitative information on the sample’s chemical profiles can help assess pyrolysis products. Extensive discussion about fragmentation routes and pyrolytic products from lignocellulosic biomass can be found elsewhere.^36–38^ Moreover, knowledge of the chemical profile of each sample can facilitate more targeted application of its bio-oil in the fuel industry.

The pyrolysis of sugar cane bagasse and straw produced bio-oil, mainly composed of phenolics, as indicated by the data presented in Table 1. Therefore, by analyzing the distribution of classes of identified peaks (Figure 2), it is possible to notice that the bagasse bio-oil presents a higher content of total phenolics.

**Figure 2:**
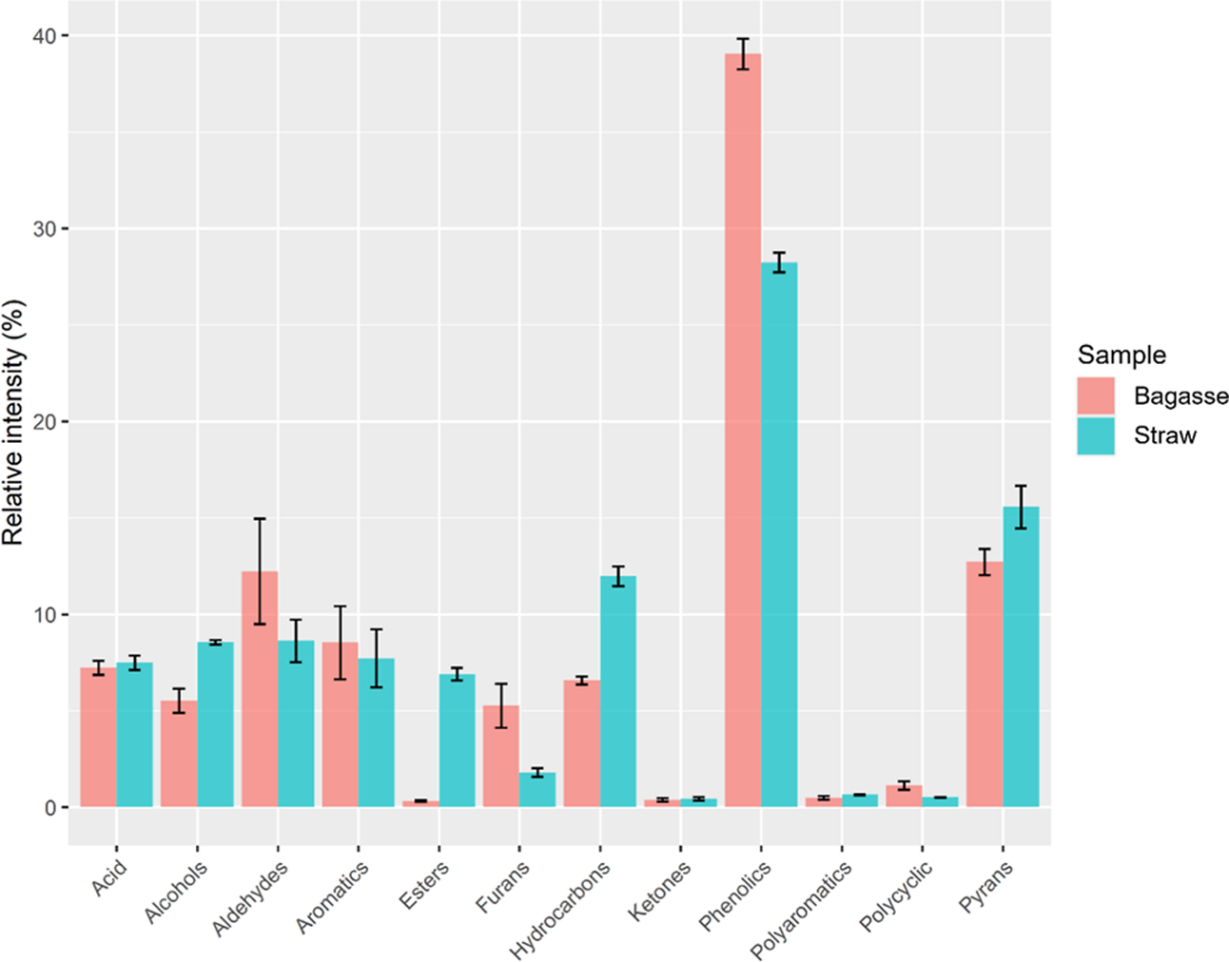
Distribution of major classes of compounds identified in sugar cane bagasse and straw bio-oil by GC×GC-TOFMS. Black bars represent the standard deviation.

Their elution profile begins with less complex structures such as benzene and phenol. The retention factor in the ^1^D increased with the molecular weight of the analyte, as observed with the increase in hydrocarbon chain length in the homologous series. More complex elution patterns are observed when increasing the number of substituents in the homologous series, as seen for the group of phenols, which are more distributed along the two-dimensional space (Figure S1). For example, we highlight phenol, guaiacol (3-methoxyphenol), and syringol (2,3-dimethoxyphenol). Conversely, sugar cane bagasse bio-oil presents more syringol, catechol, and 4-vinylphenol. Current literature suggests that sugar cane bagasse, which is composed of mature parts of the plant, has a lignin structure rich in syringol and hydroxyphenol monolignols; therefore, such fragments are expected to be identified in greater amounts in bagasse bio-oil.^39^

Regarding sugar content, sugar cane bagasse bio-oil contains higher amounts of furans and aldehydes, both carbohydrate derivatives, resulting from internal rearrangement and dehydration reactions during the pyrolysis of cellulose and hemicellulose.^40^ Among the anhydrosugars, glucopyranose, mannopyranose, and levoglucosan were prominent in both samples. Moreover, furans, such as 5-hydroxymethylfurfural, were more abundant in the bagasse bio-oil.^41^ The greater abundance of cellulose derivatives in sugar cane bagasse bio-oil, compared to sugar cane straw bio-oil, can be attributed to the higher cellulose content in the original biomass.^42^ However, levoglucosan, one of the primary cellulose pyrolysis products, showed a higher percentage of chromatographic area in bio-oil from sugar cane straw (4.8%), which is also corroborated by other studies.^15,16^ Its dehydration product, levoglucosenone, showed a contrary behavior, being more predominant in bagasse bio-oil samples.

Although hemicellulose has a similar structure to cellulose, its amorphous nature and low degree of polymerization promote thermal decomposition at lower temperatures than those required for cellulose, allowing differentiation of its fragmentation products.^43^ At 500 °C, hemicellulose mainly decomposes into noncondensable gases and oxygenated linear short-chain fragments (<C_6_), such as mono- and polyalcohols, organic acids, ketones, and aldehydes, with a minor contribution from furans and anhydrosugars.^38,43^ Therefore, such compounds can be observed at the beginning of the chromatogram as their increased polarity places them further along the ^2^D. Since sugar cane straw contains more hemicellulose, it is expected to have a greater abundance of those classes.^42^ However, data show a relatively higher intensity of these compounds in bagasse bio-oil with butanal being the most prominent, corresponding to 10.5% of the bagasse bio-oil chromatographic area. This suggests that the observed light compounds are more likely to be related to cellulose fragmentation than hemicellulose fragmentation at that temperature.

In addition, the presence of hydrocarbons and FAMEs was identified predominantly in sugar cane straw bio-oil. FAMES, such as methyl 9,12-octadecadienoate, methyl 9,11-octadecadienoate, and hexadecanoate, are present mainly in sugar cane straw bio-oil. This class of compounds may originate from the fragmentation of leaf waxes and intracellular lipids, esterification of fatty acids during pyrolysis, or natural, unaltered compounds from biomass.^8,44–48^ Undecane and dodecane were the most intense hydrocarbons in both classes of samples.

Previous characterization of sugar cane bagasse and straw bio-oils has identified phenolic contents in greater amounts, followed by aldehydes and ketones in straw bio-oil. The phenolic content was slightly higher than that found in bagasse bio-oil.^49^ Additionally, it has been reported that cellulose and hemicellulose derivatives, such as furfural and acetic acid, as well as lignin derivatives, including catechol and naphthalene, exhibit the highest intensity in sugar cane bagasse bio-oil. They also found a greater concentration of aliphatics, alkenes, and aromatics in straw bio-oil.^8,50^ Further studies employing GC–MS to analyze pyrolytic bio-oil from sugar cane straw observed acetic acid, hydroxyketone, and furfural as the most abundant compounds.^51^ Analyses combining both GC–MS and GC×GC–MS have also reported the presence of sugar derivatives, possibly due to incomplete fragmentation of cellulose, and phenolics as the most intense chemical families in straw bio-oil.^16^

### 3.2 Tile-Based Fisher Ratio for Class-to-Class Differential Features Determination

Chemometric methods allow for more efficient and accurate data processing. Tile-based Fisher-ratio analysis (FRA) was employed to uncover the differential features in bio-oil samples. The reported tiles were deconvoluted, and the resulting spectra were compared with spectral libraries. The Supporting Information (Table S2) summarizes compounds with putatively identified chemicals.

Long hydrocarbons and esters (>C_13_), such as hexatriacontane and methyl hexadecanoate, are mainly attributed to sugar cane straw bio-oil. Regarding cellulose derivatives, straw bio-oil shows higher amounts of levoglucosan and furfuryl alcohol. Additionally, benzothiazole, a compound possibly originating from the pyrolysis of sulfur-containing amino acids, is more predominant in straw bio-oil.^52^ Meanwhile, sugar cane bagasse bio-oil can be identified through furans and pyrans resulting from the thermal decomposition of hexoses, such as 5-hydroxymethylfurfural and levoglucosenone. Its lignin-derived phenolic and aromatic contents, such as syringol and 2,3-dihydro-benzofuran, can also be better described.

PCA using the unfiltered peak table of putative identifications as variables (Figure 3) showed a limited explained variance, with PC1 and PC2 accounting for 58.25% of the summed variation. This may be due to unrelated variables in the peak table, leading to high interclass variance.

**Figure 3:**
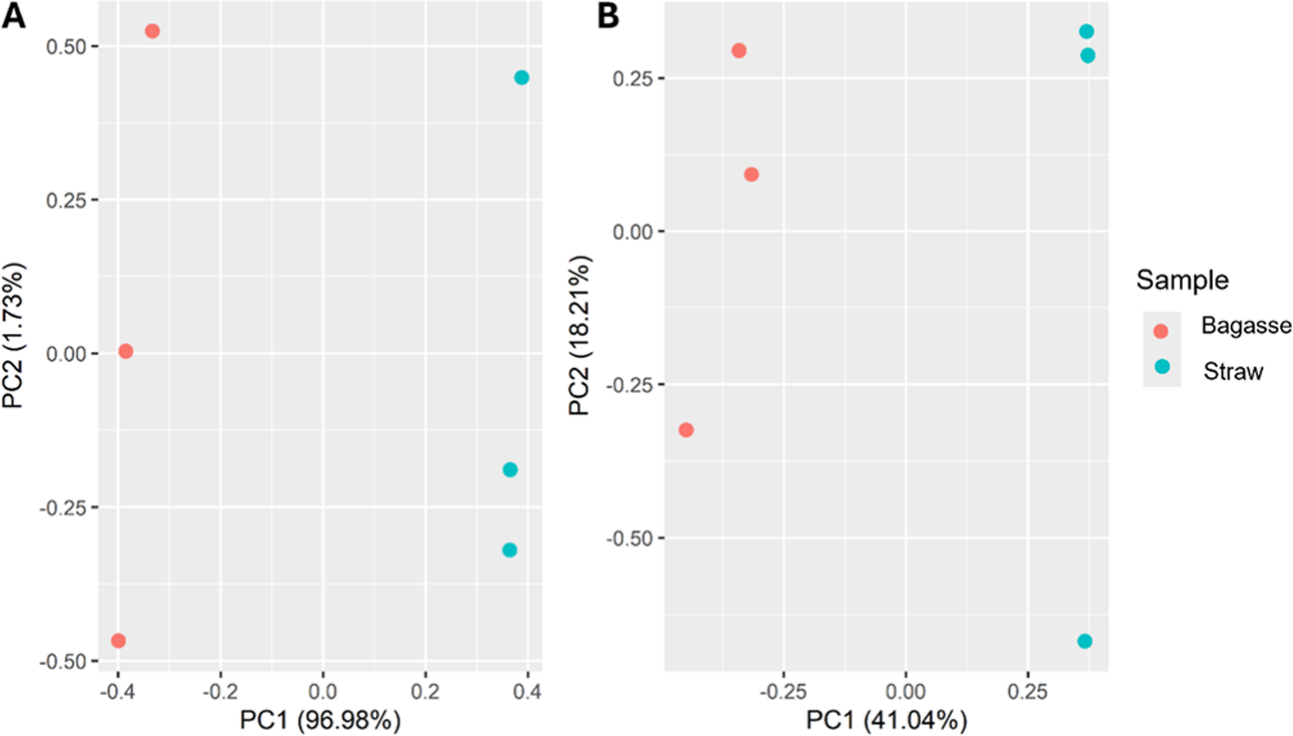
Pairwise PCA score plots of bagasse and straw bio-oil samples performed with chromatographic areas of the discriminant variables from tile-based FRA (A) and putative identifications from peak table (B).

Conversely, a PCA score plot was generated using the previously selected differential features as variables obtained through FRA. The data were mean-centered and Pareto-scaled before analysis. Figure 3A shows the pairwise PCA of bagasse and straw bio-oils and replicates. A distinct clustering of the groups, as described by PC1, explains 96.98% of the variation. Since the data matrix consists of two samples and replicates, PC1 groups samples by their biomass origin, while PC2 (1.73% of variation explained) represents technical variability among replicates, as suggested by Sudol et al.^53^

The tile-based approach filters all chromatogram information without requiring deconvolution, alignment, or prior identification. As a result, this method enables the detection and discovery of analytes by FRA at lower intensities than the regular peak table-based approach. For example, 5-hydroxymethylfurfural was identified exclusively in the straw bio-oil samples through tile processing. Sixteen compounds were determined only by tile-based approach in at least one of the samples, including methyl octadecenoate, octacosane, hexacosane, and tetracosane. The intensities of these features ranged from 5.5 × 10^4^ to 3.8 × 10^7^, suggesting the limited detection capabilities of the deconvolution algorithm used in the peak table-based approach. Moreover, this tile strategy provides a more representative sample profile by evaluating differential features across the entire chromatogram rather than relying solely on the identified peaks. Therefore, the tile-based FRA for selecting the most discriminative variables provides more informative features for describing the bio-oil chemical composition and its origin.

Such findings can contribute to improved bio-oil chemical profiling and propose more appropriate industrial applications. For example, the identified content of hydrocarbons and esters contributes to a higher heating value for bio-oil, which is advantageous for applications in the energy sector.^8^ However, the length of hydrocarbon chains increases the viscosity and deposition, which is not ideal for fuel or additive applications. Furthermore, the acidic products from hexoses and fatty acid fragmentation lower the pH and render bio-oil unsuitable as a fuel or additive. Additionally, the acids can react with other compounds in bio-oil, producing unexpected byproducts during production and storage.^38,54^ To overcome such problems, sugar cane straw bio-oil should undergo upgrading reactions to increase hydrocarbon production and reduce oxygen content in the sample through hydrodeoxygenation, for example.^55^

## 4 Conclusions

The analytical strategy enabled the characterization of bio-oil samples from sugar cane bagasse and straw using GC×GC–MS and chemometrics. Both bio-oils presented a high content of lignin derivatives, with the bagasse pyrolytic bio-oil exhibiting the highest content of phenolics. Regarding hexose derivatives, furans and aldehydes were mainly found in bagasse bio-oil due to the higher hemicellulose content in the biomass. Straw bio-oil showed significant amounts of hydrocarbons and esters from C_11_ to C_19_ due to the higher lipid content from leaf waxes and intracellular lipids. Applying the chemometric approach, tile-based FRA enabled the identification of discriminant features that better describe each sample in terms of its biomass origin and chemical composition, allowing the differentiation of samples with similar chromatographic profiles. Additionally, the approach enabled the identification of low-intensity and coeluted compounds that were not identified by a regular deconvolution algorithm, such as very long hydrocarbons and esters (>C_19_). Tile-based FRA has been demonstrated to be a more representative method for variable selection and sample group discrimination when dealing with the complex data generated by GC×GC-TOFMS analysis, assisting in the chemical understanding of bioproducts for industrial applications.

## Supplementary Material



## Notes

Putatively identified compounds through peak table approach for sugar cane straw and bagasse bio-oil; hitlist for tile-based FRA differential features; and apex plot of substituted phenols, methoxyphenols, and dimethoxyphenols (XLSX)
